# Estimate of undergraduate university student alcohol use in China: a systematic review and meta-analysis

**DOI:** 10.1186/s13690-017-0220-x

**Published:** 2017-12-11

**Authors:** Ian Newman, Lanyan Ding, Yonghua Feng

**Affiliations:** Nebraska Prevention Center for Alcohol and Drug Abuse, Department of Educational Psychology, University of Nebraska-Lincoln, PO Box 880345, Lincoln, NE 68588-0345 USA

**Keywords:** Alcohol, China, Drinking, University students, Meta-analysis

## Abstract

**Objective:**

To develop an estimate of self-reported last 30 day alcohol use by university students in China.

**Methods:**

A search of papers published in English and Chinese between 2006 and 2015, following pre-established selection criteria, identified 30 papers that were included in this meta-analysis. Nine moderator variables were preselected for this analysis.

**Results:**

A total of 749 papers were identified in the keyword search, and 30 studies (28 in Chinese, 2 in English) met all selection criteria and were included in the meta-analysis. The self-reported last-30-day alcohol use for undergraduate university students was 66.8% for males and 31.7% for females. Meta-regression identified three moderators associated with the different drinking rates reported: the definition of drinking, the origin of the questionnaire used in the survey, and the geographic region where the survey was conducted. These three moderators explained 56% of the heterogeneity of reported drinking rates for the male students and 47% of the heterogeneity of reported drinking rates for the female students.

**Conclusions:**

The results of this meta-analysis provide an estimate of last 30 day alcohol use by university students (age 18–23) and increase our understanding of drinking by young people in China. The meta-analysis suggested three variables that could have affected the results and which are worthy of further study. The discussion places these results in the context of Chinese drinking culture and university life.

## Introduction

The World Health Organization (WHO) suggests a 36% increase in per capita alcohol consumption in liters of pure alcohol in China between 2003–2005 and 2008–2010 [1]. In 2014, WHO estimated that 58.4% of adult males and 28.9% of adult females in China had consumed alcohol in 2010 [2]. While these data and others from large national surveys among different populations [3–8] provide the best estimates of drinking patterns, there are few studies that describe drinking among the younger population. Data from the younger population has the potential to suggest something about trajectory towards these adult drinking patterns [9]. For example, a recent meta-analysis of studies published between 2007 and 2015 of drinking rates of Chinese adolescents in middle school (ages 13–15) and high-school (ages 16–18) suggests that 36.5% of the males and 21.2% of the females in high school had consumed alcohol in the previous 30 days as had 23.6% of the males and 15.3% of the females in middle schools. The highest drinking rates—44.7% of the males and 28.8% of the females— were among vocational high school students (students not intending to take the national college entry exam or study at university) [10].

To further explore the trajectory of adolescent to adult drinking, this project was developed to analyze last 30 day alcohol use among Chinese undergraduate university students that were reported in papers published between 2006 and 2015 in English and Chinese.

Students who enter university in China follow a specific educational path. The nation provides compulsory general education through middle school (age 15). At the completion of middle school, university-bound students enter a regular high school, and the other students enter a vocational high school. Vocational high schools prepare students with the technical skills to directly enter the workforce after 2 to 3 years. The regular high school students, meanwhile, attend high school for three years and take the national university entrance exam (*gaokao*) at the end of their 3rd year. This is a highly competitive educational path, and the *gaokao* result determines which universities will accept a student’s application for admission. The higher the student’s score on the *gaokao*, the greater his/her chance of being admitted to one of the more prestigious universities. Students with lower scores will apply to less prestigious universities or to community colleges/city colleges. The community/city college option results in a three-year degree, the university option results in a four-year degree.

Entering university represents a significant lifestyle change for most students. The new environment places new demands for self-regulation in the absence of family regulation. Western research literature describes the beginning of university as a time of experimentation often associated with increased alcohol use [11]. Our research group is interested in understanding alcohol use in different life stages—from adolescence to adulthood. This meta-analysis focused on studies of alcohol use by Chinese undergraduate university students, who typically are 18–23 years old. This paper complements an earlier meta-analysis of studies of alcohol use among Chinese middle and high school students [10].

Two research questions guided this study:

1. What is the 30-day drinking rate for male Chinese undergraduate university students?
2. What is the 30-day drinking rate for female Chinese undergraduate university students?

## Method

### Research protocol

The Preferred Reporting Items for Systematic Reviews and Meta-Analysis (PRISMA) guided this project (http://www.prisma-statement.org/PRISMAStatement/PRISMAStatement.aspx).

### Information sources search strategy

We conducted an online search of four databases: China National Knowledge Infrastructure database (CNKI) [12], Wanfang (WF) [13], PubMed (PM) [14], and Web of Science (WS) [15]. In each database we searched for papers published between 2006 and 2015. Initially we searched for titles and abstracts containing the keywords: college students/university students AND alcohol use/drinking AND China/Chinese.

### Eligibility Criteria

Five pre-established criteria were determined for a study’s inclusion in the analysis. Papers had to 1) be based on original data, 2) report the last 30-day drinking rate, 3) report drinking rates by gender, 4) describe students attending a university/college in Mainland China, 5) be published in a peer-reviewed journal in English or Chinese between 2006 and 2015.

#### Original data

To increase the integrity of the data used in the meta-analysis we selected only papers reporting on original survey data. Articles using duplicated data or secondary sources were excluded.

#### Last 30-day drinking rate

Previous studies have estimated drinking rate in the past seven days, 30 days, three months, six months and last year. We selected the past 30 day recall because it was the most frequently used, because it allowed sufficient time to capture drinking behaviors, and because it was less subject to memory error than longer recall periods [16]. Alcohol quantity data were not used in this meta-analysis. Self-reported estimates of alcohol quantity in China are confounded by a number of factors such as the absence of a standardized drink size, wide variation in the strength of beverage alcohols, access to unlabeled home-made and unrecorded alcohols, drinking from different sizes of cups and bowls, and a variety of drinking customs [17–19].

#### Gender

Previous surveys of drinking behavior in China have suggested there are significant differences in the drinking rates of males and females [1, 3, 18, 20]. These gender differences reflect social attitudes about gender roles and drinking [21]. Both national and regional studies of alcohol consumption among Chinese university students have indicated gender differences [8, 9].

#### Mainland China

Hong Kong, Macau and Taiwan were excluded because their recent history differs significantly from that of the Mainland. Studies of Chinese students in other countries were excluded.

#### Published in English or Chinese

We recognize there may be relevant studies published in other languages, but we lacked the resources to seek them out.

### Methodology quality assessment

The search of the two Chinese databases and two American databases was done twice by one reviewer to ensure we captured all of the possible studies relating to our research questions. Eligibility for inclusion was assessed by two reviewers independently. Any disagreements between the two reviewers were discussed until consensus was reached. If there was no consensus, the paper was excluded. To ensure the methodological quality of the papers included in this meta-analysis, the guidance tool *Quality Assessment of Systematic Reviews and Meta-Analyses* (NIH) was used [22]. Two reviewers independently rated the quality on each of eight items listed in this quality assessment scale from good, fair, to poor. Data extractions was carried out by two researchers independently and the results were compared. Publication bias and heterogeneity were assessed in a meta-analysis.

### Outcome variables and moderator variables

The outcome variable for this study was the logit of the self-reported drinking rates of male and female undergraduate students in China.

The importance of moderator variables in meta-analyses has been noted by Groves and Lyberg [23], Moher et al. [24] and Feng and Newman [10]. Based on their recommendations and the comments from Chinese and American researchers who had considerable experience studying alcohol use in China, nine moderator variables were identified. They were: 1) year the study was conducted, 2) the number of universities surveyed, 3) the response rate, 4) the origin of the alcohol use questionnaire, 5) a clearly stated definition of drinking as having at least one cup of alcohol beverage, 6) trained or untrained data collectors, 7) four-year or three-year university degree program, 8) the geographic region of the study, and 9) the sample size.

The moderator variable for origin of the alcohol use questionnaire was identified for the following reason: Often, in emerging areas of investigation, the survey questions are developed by individual investigators. One earlier attempt to summarize findings from studies done between 1994 and 2004 of university student drinking in China failed partly because of the great diversity of investigator-developed alcohol use questions [9]. In contrast to that earlier attempt, this study found that 20 of the 30 papers identified contained alcohol use questions based on the Youth Risk Behavior Survey (YRBS). The YRBS was developed by the US Centers for Disease Control and Prevention and first administered in the USA in 1991. The YRBS aimed to standardize data collection about epidemiologically identified health risk factors of adolescents. The questions have been refined through frequent use and revision; consequently the questions tend to be more specific. The YRBS questionnaire continues to be used in the United States and has influenced adolescent health surveys worldwide with its standardized questions of clearly-defined behaviors. The contribution of the YRBS to the improvement of survey instruments is evidenced in this collection of papers. Judging by this sample of alcohol use studies in China, it appears that beginning in 2006 standardized alcohol use questionnaires based on the YRBS began replacing investigator-developed alcohol use questionnaires.

### Moderator coding

The year the study was conducted, the number of schools surveyed, and the response rate were treated as continuous variables. The origin of alcohol use questionnaire was dummy coded as Youth Risk Behavior Survey (YRBS) =0, investigator-developed questionnaire =1. Whether or not the definition of drinking was specified and whether the data collectors were trained were both dummy coded as No = 0 and Yes = 1. The university/college type was coded as 1 = 4-year university, 2 = 3-year college, and 3 = combined results from a 3-year college and 4-year university or missing. The geographic region was coded into two moderators— east = 1, central = 2, and west = 3, then south = 1 and north = 2 —according to the classification determined by the National Bureau of Standards of China [25]. Studies conducted across more than one geographic region were coded as missing. The sample size was coded into three percentiles: 0–33rd percentile =1, 34th–66th percentile =2, and >67th percentile =3.

### Publication bias

Funnel plots presented visual symmetry of the outcome variables. Egger’s linear regression test quantified the bias represented in the funnel plot allowing a test for significance [26].

### Heterogeneity

Heterogeneity refers to the degree of between-study variance due to true effect rather than chance and is indicated as I^2^. A value of 0% indicates no observed heterogeneity; higher values indicated greater heterogeneity [27]. Cochran’s Q-test was used to determine if the differences in outcome estimates across studies were larger than expected by chance. A significant Q-value indicates heterogeneity.

### Sensitivity analysis

To further address heterogeneity concerns a sensitivity analysis was conducted by omitting one study at a time and re-examining the overall results.

### Meta-analysis

DerSimonian and Laird random effect model was used to estimate pooled drinking rates [28]. This model is recommended when it is expected that different studies will have different effects [29]. The model assigns heavier weights to larger studies [30].

### Meta-regression

Meta-regression with maximum likelihood estimation was used to explore the effects of the moderator variables associated with the true between-study variance (I^2^). R^2^ represented the between-study variance explained by the moderators. The log transformed value of ratio outcome variable was used to generate a symmetric scale with a symmetric confidence interval [31]. To ensure that the analysis would be meaningful, the recommended ratio of studies to moderators is at least 10:1 for meta-regression [29]. Considering the small numbers included in this analysis, a bivariate meta-regression was first conducted to identify which moderators may have significant effects on the logit drinking rate. The bivariate meta-regression significance level was set at .05. All the significant moderators were then included in a multiple meta-regression model to assess the relationship between multiple moderators and the logit drinking rate [29]. Since the statistical power tends to be reduced due to the small sample size of included studies, the significance level was set at *p* = .1 for the multiple meta-regression model [10, 31]. To avoid the inflated likelihood of error in the multiple meta-regression analysis, Bonferroni correction was used to adjust the *p*-value as .033 for a three-moderators model [32].

### Subgroup analysis

Subgroup analysis (analogous to analysis of variance) was applied to categorical moderators that were significantly associated with the heterogeneity identified in the multiple meta-regression analysis. The pooled drinking rate for subgroups was estimated with the random effect model. A Q-value with *p* < .05 indicates a significant group difference in last 30 day alcohol use.

Comprehensive Meta-analysis Professional Version 3 was used for the data analysis. The significance level was set at two-sided *p* = .05 for all analyses except for the multiple meta-regression analysis with adjusted significance level of *p* = .03. A list-wise deletion method was used for missing data.

## Results

**Fig. 1:**
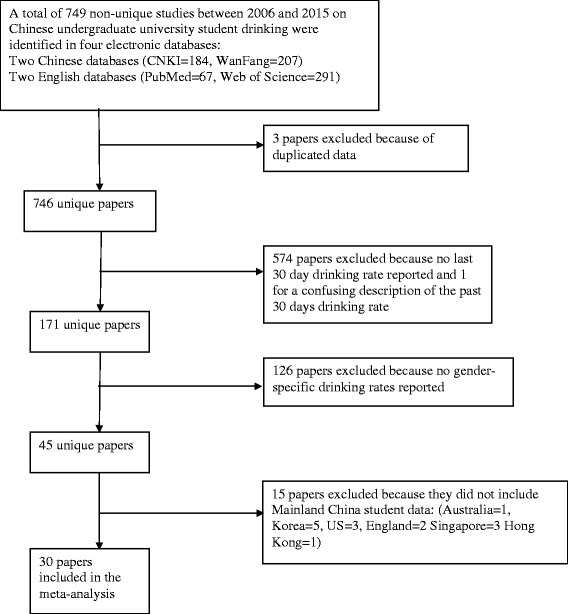
Flowchart of study selection, inclusion and exclusion for surveys of Chinese university student past-30-day alcohol use, 2006–2015. CNKI = China National Knowledge Infrastructure, WF = Wanfang, PM = PubMed, WS = Web of Science

A total of 749 papers were initially identified through keyword search: (184 in CNKI, 207 in WF, 67 in PM, 291 in WS). Studies in WF that overlapped with studies first identified in CNKI were not counted. Similarly, studies identified in WS that had already been found in PM were not counted. Three papers reported on duplicated data and were excluded. Of the remaining 746 papers, 574 were excluded because they did not report on past 30 day alcohol use; and one study was excluded due to a confusing description of the past 30 day drinking rate. Of the remaining 171, 126 were excluded because they did not report gender-specific drinking rates. Fifteen papers that did not report Mainland China drinking studies were excluded. The remaining 30 studies (28 in Chinese and 2 in English) were included in this analysis (Table 1). A flowchart is provided in Fig. 1 for the inclusion and exclusion process.

**Table 1:** Characteristics of the studies from 2006 to 2015 included in the meta-analysis of last-30-day alcohol use of Chinese university undergraduate students

Reference	Pub year	Sample size	Drinking rate in past 30 days (%)			Alcohol Use Questionnaire	Response Rate (%)	Trained data collector	Drink definition	School *N*	University type	Province	East vs. Middle vs. West	North vs. South
			Males	Females	Total									
Peng et al. [33]	2006	1765	57.90	30.60	45.40	YRBS	98.33	no	yes	7	combined	Shanghai	East	South
Sun [34]	2006	1843	69.60	35.30	48.00	YRBS	96.70^T^	yes	yes	5	4-year	Sichuan	West	South
Aximu [35]	2007	609	51.30	30.8	42.36	YRBS	N/A	yes	no	N/A	N/A	Xinjiang	West	North
Yin [36]	2007	942	65.40	43.00	54.00	YRBS	96.10	no	yes	2	3-year	Jingzhou	Middle	South
Zhai [37]	2007	1138	30.50	7.50	17.05	YRBS	N/A	yes	yes	2	4-year	Beijing	East	North
J. Zhang et al. [38]	2007	442	44.60	11.20	20.5	ID	N/A	no	no	1	4-year	N/A	N/A	N/A
Zhong [39]	2007	2319	69.00	29.50	50.00	YRBS	N/A	yes	no	8	N/A	Henan	Middle	North
Liang et al. [40]	2008	1752	81.24	35.06	58.39	ID	97.30	yes	no	3	4-year	Baotou	West	North
Ruan et al. [41]	2009	4013	64.20	31.20	44.50	YRBS	99.45^a^	yes	yes	4	N/A	Guangxi	West	South
Z. Wang et al. [42]	2009	3051	37.11	12.13	26.12	ID	99.18^a^	no	yes	8	combined	Beijing	East	North
Yu [43]	2009	978	84.63	51.30	71.47	ID	97.80	no	no	5	4-year	Liaoning	East	North
Zhu et al. [44]	2009	615	71.60	41.67	53.50	YRBS	97.43^a^	no	no	2	N/A	Gansu	West	North
Dai et al. [45]	2010	1025	46.20	22.20	34.1	YRBS	93.20	yes	no	1	4-year	Guangdong	East	South
Guo & Liu [46]	2010	992	82.50	37.90	54.60	YRBS	99.20	yes	no	2	3-year	Shandong	East	North
Lin [47]	2010	686	65.20	38.60	53.94	YRBS	99.88^T^	yes	yes	2	N/A	Fujian	East	South
C. Zhang et al. [48]	2010	2126	41.60	18.40	30.90	YRBS	96.64	yes	yes	6	combined	Qinhuangdao	East	North
T. Zhang et al. [49]	2010	576	68.60	30.80	51.40	YRBS	100.00	yes	yes	2	4-year	Chengdu	West	South
Liu et al. [50]	2011	397	98.25	80.96	90.93	ID	88.22	no	no	5	N/A	Mongolia Baotou	West	North
Cao et al. [51]	2012	1807	72.21	37.80	54.17	YRBS	N/A	yes	yes	N/A	N/A	Qinghai	West	North
Ji et al. [8]	2012	51,250	66.60	34.70	49.30	YRBS	81.40	no	yes	119	combined	National	N/A	N/A
L. Xu [52]	2012	516	59.50	28.40	41.10	YRBS	100.00	yes	yes	1	4-year	Shan’xi	West	North
Guo et al. [53]	2013	7979	58.60	26.90	42.20	YRBS	N/A	no	yes	44	combined	National	N/A	N/A
Newman, Jinnai, et al. [54]	2013	725	60.77	29.02	43.86	ID	67.10	yes	no	4	N/A	Beijing, Ji’nan, Wuhan, Xianning	N/A	N/A
X. Xu et al. [55]	2013	1437	56.50	23.00	38.00	YRBS	88.50	yes	yes	3	combined	Jiangsu Nantong	East	South
R. Zhang [56]	2013	1968	62.00	29.80	41.92	YRBS	N/A	yes	no	6	4-year	Zhejiang	East	South
Newman, Huang, et al. [57]	2014	530	62.50	26.00	39.20	ID	90.30	yes	no	4	N/A	Beijing, Zhengzhou	N/A	North
Wan [58]	2014	492	94.30	56.20	72.56	ID	94.60	yes	no	1	4-year	Hun’an	Middle	South
Y. Zhang et al. [59]	2014	1044	61.30	26.20	40.23	YRBS	99.73^T^	yes	yes	3	3-year	Jiangsu	East	South
Du et al. [60]	2015	1452	58.70	20.20	33.10	ID	96.80	no	yes	3	4-year	Qinghai	West	North
C. Wang et al. [61]	2015	2849	92.30	68.80	80.20	ID	95.00	yes	yes	8	combined	Anhui Hefei	Middle	South

^a^Some studies were not limited to only university students. In these cases the response rate included their total sample rather than just the university students

**Fig. 2:**
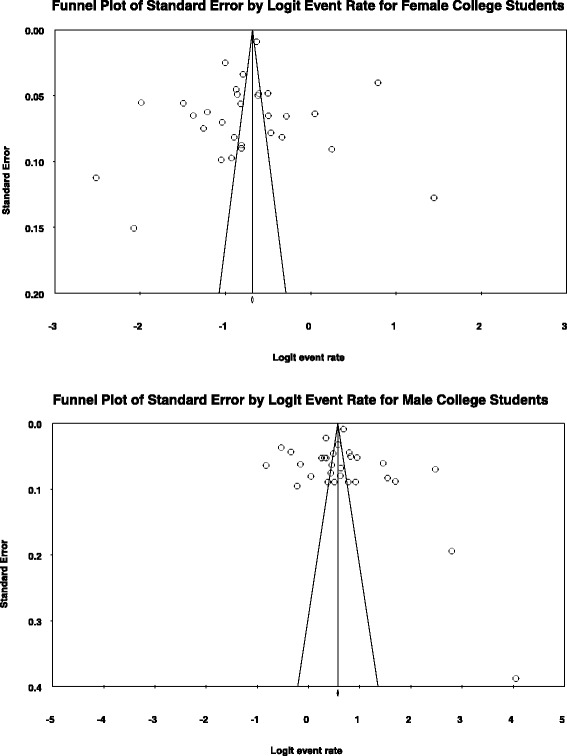
Funnel plots of estimated past 30 day alcohol use from surveys of male and female university students in China between 2006 and 2015 using the DerSimonian-Laird random-effect model. *Note*. Egger’s regression test (2-tailed) for publication bias for male university students’ drinking rate in the past 30 days, t (28) = .14, standard error = 3.20, *p* = .89. *Note*. Egger’s regression test (2-tailed) for publication bias for female university students’ drinking rate in the past 30 days, *t* (28) = .70, standard error = 3.02, *p* = .49

The funnel plots for both males and females were symmetric. Eggers’s linear regression test reported a two-tailed nonsignificant *p*-value for males (*p* = .889) and for females (*p* = .488,) indicating no publication bias (Fig. 2).

**Fig. 3:**
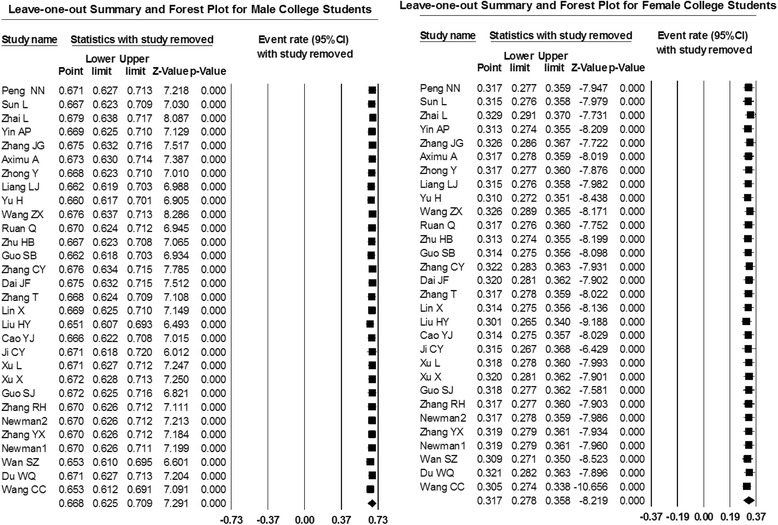
Sensitivity analysis summary and forest plots for Chinese university student drinking surveys from 2006 to 2015 using the DerSimonian-Laird random-effect model

The sensitivity analyses, which involved omitting one study at a time and recalculating the overall results, suggested that for both male and female university student alcohol use were consistent, suggesting that no individual study significantly affected the findings (Fig. 3).

### Male university students alcohol use

The pooled estimate of drinking among male university students in the last 30 days was 66.8%, 95% CI [62.5, 70.9] (Table 2). There was evidence of heterogeneity among the selected 30 studies (I^2^ = 99.26; Q = 3940.25, df = 29, *p* < .001).

**Table 2:** Pooled estimates of drinking in the past 30 days for male and female university students, data from China 2006–2015

Subgroup	*N*	Rate range (%)		Pooled rate (%)	95% CI		*Q*	I^2^ (%)
		LL	UL		LL	UL		
Male	30	30.50	98.25	66.8	62.5	70.9	3940.25^***^	99.26
Female	30	7.5	80.96	31.7	27.8	35.8	3566.0^***^	99.19

*CI* confidence interval, *LL* lower limit, *UL* upper limit

^***^ *p* < .001 for the Cochrane *Q* test

According to the results from the bivariate meta-regression analysis, the definition of drinking, the origin of the questionnaire, and the geographic region of the survey were associated with the heterogeneity across the selected studies on the logit male drinking rate (Table 3). Surveys with stated definitions of drinking reported lower drinking rates than surveys without a stated definition (*β* = −.8216, *p* < .01). The moderator of definition of drinking explained 20% of the heterogeneity. Surveys that used investigator-developed questionnaires reported higher drinking rates for males than surveys based on the more standardized YRBS questionnaire (*β* = .9754, *p* < .01), and the moderator for questionnaire explained 21% of the heterogeneity. Surveys of central China university students reported higher drinking rates than surveys of east China university students (*β* = 1.0885, *p* < .01). Surveys of west China university students reported higher drinking rates than surveys of east China university students (*β* = .7360, *p* < .05). The moderator for geographic region (east, central, west) explained 21% of the heterogeneity.

**Table 3:** Moderators associated with the heterogeneity of past-30-day drinking rate estimates in surveys of Chinese university students, 2006–2015

Outcome variables	Moderators	Bivariate *β*	SE	R^2^%	Multiple Meta-regression *β*	SE	R^2^%
Logit drinking rate of males							
	Definition (yes = 1, no = 0)	−.8216^**^	.3060	20	−.7886^**^	.297	56
	Questionnaire (YRBS = 0, Investigator-developed = 1)	.9754^**^	.3492	21	.8795^**^	.341	
	Region (east to central)	1.0885^**^	.4817	21	.711^*^	.3762	
	Region (east to west)	.7360^*^	.4021		.6184^*^	.317	
Logit drinking rate of females							
	Definition (yes = 1, no = 0)	−.5670^*^	.2595	14	−.6633^**^	.2703	47
	Questionnaire (YRBS = 0, Investigator-developed = 1)	.5892^*^	.3018	12	.3919	.2975	
	Region (east to central)	.8886^**^	.3744	22	.5847	.3302	
	Region (east to west)	.6017^*^	.3121		.5242	.2747	

^**^ *p* < .01,^*^ *p* < .05 for bivariate regression. Bonferroni correction applied on the significance level of .1 for multiple meta-regression analysis: the significance level = .1/3 = .033 for 3 moderators

A multiple meta-regression model was used to assess all the significant moderators in one meta-regression model. The variables of a clearly stated definition of drinking, the origin of questionnaire, and geographic region were still significant after the Bonferroni correction and explained 56% of the heterogeneity in male drinking rate.

Considering the significant effect of three categorical moderators (definition of drinking, origin of questionnaire, and geographic region) on the heterogeneity, a follow up test was performed to determine whether the pooled drinking rate for male university students differs between groups. In the subgroup analysis, the pooled drinking rate for male students was 70% using definition of alcohol drinking and 63% without definition. A significant Q value indicates a significant group difference in the logit drinking rate of males (Q (1) = 227.12, *p* < .001). The pooled drinking rate for male students was 64% for using YRBS questionnaire and 60.1% for using investigator-developed questionnaire. The pooled drinking rate for male students in the east, central and west regions was 52.1%, 74.8%, and 67.5% respectively (Q (2) = 1252.67, p. < 001). The group difference is significant for the logit male drinking rate (Q (1) = 257.30, *p* < .001) (Table 4).

**Table 4:** Pooled estimates for subgroups using DerSimonian-Laird Random-Effect Model for Chinese university students’ past-30-day alcohol use, 2006–2015

Outcome variable	Subgroup	*N*	Pooled estimate(%)	95% CI	I^2^	Q_between_
Pooled male drinking rate						
	Definition (no)	13	70.0	69.2–70.8	99.17	227.12^***^
	Definition (yes)	17	63.0	62.7–63.4	99.31	
	Questionnaire: (YRBS)	21	64.0	63.7–64.3	98.84	257.30^***^
	Investigator-developed	8	60.1	59.0–61.3	99.64	
	Region east	11	52.1	51.3–52.9	99.29	1252.67^***^
	Region middle	5	74.8	73.7–75.9	99.35	
	Region west	9	67.5	66.6–68.3	97.92	
Pooled female drinking rate						
	Definition (no)	13	42.5	41.6–43.4	99.26	492.86^***^
	Definition (yes)	17	32.2	31.9–32.5	98.89	

*YRBS* Youth Risk Behavior Survey

^***^ *p < .001*

### Female university students alcohol use

The pooled estimates for the drinking rate in the past 30 days among female university students was 31.7%, 95% CI [27.8, 35.8] (Table 2). There is evidence of heterogeneity (I^2^ = 99.19, Q = 3566.0, df = 29, *p* < .001) among reported drinking rates.

According to the results of the bivariate mega-regression analysis, the definition of drinking, the origin of the questionnaire, and the geographic region of the survey were associated with the heterogeneity across the selected studies on the logit female drinking rate (Table 3). Surveys with stated definitions of drinking reported lower drinking rates than surveys without a stated definition (*β* = −.5670, *p* < .05). The moderator of definition of drinking explained 14% of the heterogeneity. Surveys that used investigator-developed questionnaires reported higher drinking rates than surveys based on the more standardized YRBS questionnaire (*β* = .5892, *p* < .05), and the moderator for questionnaire explained 12% of the heterogeneity. Surveys of central China university students reported higher drinking rates than surveys of east China university students (*β* = .8886, *p* < .01). Surveys of west China university students reported higher drinking rates than surveys of east China university students (*β* = .6017, *p* < .05). The moderator for geographic region (east, central, west) explained 22% of the heterogeneity.

Results from the multiple meta-regression analysis indicated that only the variable ‘a stated definition of alcohol drinking’ is significant after the Bonferroni correction, and this variable explained 47% of the heterogeneity.

A subgroup analysis was applied to the significant categorical variable of definition identified in the meta-regression analysis. In the subgroup analysis, the pooled drinking rate for female students was 42.5% using definition of alcohol drinking and 32.2% without definition. A significant Q value indicates significant group difference (Q (1) = 492.86, *p* < .001) (Table 4).

## Discussion

This meta-analysis provided an estimate of Chinese undergraduate university student drinking rates in the last 30 days of 66.8% for male university students and 31.7% for female university students. As expected, the estimated drinking rate is higher for males than for females. While many papers on alcohol use by university students have been published, this is the first to present an estimate of last 30 day drinking rates based on surveys of Mainland China undergraduate university students, published in English or Chinese, and describing male and female alcohol use separately. The results reflect the best estimate of last-30-day alcohol use by Chinese undergraduate university students.

This estimate for university students’ alcohol use is higher than WHO’s estimates of per capita drinking in the previous year for persons 15 years and older in China: 58.4% male, 28.9% females [1, 2]. We will refrain from offering explanations of why university undergraduate student drinking rates appear to be higher than adult per capita drinking rates. That question is beyond the scope of this analysis.

This estimate for university students’ alcohol use is also higher than the last 30 day drinking rates estimated for high school students: 36.5% males and 22.2% females attending regular high schools, and 44.7% males and 28.8% females attending vocational high schools [10]. The significant differences between high school drinking rates and the university student drinking rates could be a direct result of the way Chinese high school students focus on preparation for the university entrance examination (known as the *gaokao*).

Studies of Chinese high school students’ drinking have found that vocational high school students report higher drinking rates than regular high school students [10]. One of the main differences between the two types of high school students is that regular high school students are preparing for the *gaokao,* while the vocational high school students typically are not. Preparation for the *gaokao* is rigorous and begins before the last year of high school. Students pressure themselves to a strict discipline of study, reinforced and supervised by parents and teachers. There is little time for any recreational activities. By comparison the vocational high school students have more discretionary time to be involved in activities that include alcohol. Once high school students have finished the g*aokao* and have been accepted to universities, they are under much less pressure and have more freedom to engage in activities that could include alcohol. In this manner the *gaokao* may be protective for high school students by delaying their drinking until they enter university. There are likely other individual-specific explanations for these rates that have not been identified and studied.

The university student drinking rates need to be interpreted in the context of Chinese university life and Chinese alcohol culture. China is a relationship society, meaning the social structure is based first on relationships. Actions that build, maintain and protect relationships are important, and sharing alcohol is an important part of this process. Alcohol use in moderation is considered good for health, it is a part of meals, ceremonies and celebrations, and it is an important part of Chinese medicine. It is legal for university students to purchase and consume alcohol. Chinese universities, with the exception of the newest campuses, are walled compounds with everything inside the walls that students need: housing, recreational facilities, food services, health services, educational services, post offices, small shops and banks. During the week there is little need to leave the campus. On weekends students will often go off-campus with friends to eat a lunch or dinner at local restaurants. Eating with friends is a typical occasion for drinking alcohol, usually beer. In this context, the last-30-day rates reported here are not surprising and do not necessarily represent high risk drinking.

The moderator analysis showed that the heterogeneity observed affected the results, and the significant I ^2^ suggests caution in interpreting these results. Nine moderator variables were examined in the meta regression, and three of the moderators should be considered in interpreting these results: whether the survey stated a definition of drinking, whether the questionnaire was based on YRBS or was investigator developed, and the geographic region where the data were collected.

### Definition of drinking

A clearly stated definition of drinking as having “at least one cup” of an alcohol beverage (yes vs. no) was associated with the logit drinking rates of male and female university students. Surveys with stated definitions of drinking reported lower drinking rates compared to surveys without definitions (Table 4). This finding is similar to one reported in an earlier meta-analysis of high school student drinking rates [10]. In published studies of Chinese alcohol use, surveys have used drinking self-report for the past week, past 30 days, past 3 months, past 6 months, past year, and lifetime. Even in surveys that specify the past 30 days, alcohol questions differ on the basis of quantity. Some questions ask if a respondent has drunk alcohol at least once, including one sip, some questions specify at least one cup. Specificity has its value in quantifying a behavior, but it is also possible that specificity overlooks important drinking patterns that deserve attention. There is a need for a standardized definition of alcohol drinking to improve the accuracy of estimating drinking rates [9]. This standardized definition needs to be based on a careful observation of actual student drinking behaviors within Chinese alcohol culture.

### Questionnaire development

Logit drinking rates for males were related to whether or not the questionnaire was developed by the investigator or based on YRBS. Studies that used YRBS-based questionnaires reported higher drinking rates for males compared to studies using investigator-developed questionnaires. There was no significant difference in female last-30-day drinking based on type of questionnaire (Table 4). We believe men’s drinking tends to be more nuanced, with drinking varying according to traditional, established protocols regarding appropriate drinking for the time, place, occasion, and companions. In China, women drinking in public is relatively new. That is not to say that women did not drink on many occasions out of public view, often only in the company of other women; however, these occasions tended to be traditional functions like rites of passage, lunar festivals, and other special ceremonies where drinking occurred as part of the ceremony. Now there is a wider acceptance of women drinking in public, an awareness of more female drinking, and an acknowledgement of female-only drinking occasions. The relative newness of acceptability of female drinking may mean its repertoire is limited and either type of questionnaire captures the behavior equally well. Men’s drinking behavior, while superficially standardized, because it is so widespread and so integrated into daily life may be better captured by investigator-developed questionnaires than by the more standardized (restricted) YRBS-type alcohol use questions.

The most likely variations in questionnaire development that affect these differences relate to 1) how alcohol is defined, 2) how quantity is measured, and 3) how frequency is measured, and 4) the way Western questions are translated into Chinese.

#### Definition of “alcohol”

In the West, alcohol questionnaires are generally organized around three types of alcohol: beer, wine, and spirits. In China, the classification of alcohol types is more complicated: there are more types of beverage alcohol and there is no widely accepted understanding of a standard drink. Beer is comparable, with beer strength and packaging being fairly similar in China and the West. In China “wine” can mean fruit wines (often imported, Western-style strength and packaging), but it also refers to traditional wines such as *huangjiu* (yellow wine, common in the Shanghai area) or low strength rice wines and porridges. Distilled spirits are popularly classified by the grains used to make them and by their strength (high-strength spirits and low-strength spirits, with about 40% alcohol-by-volume being the threshold). Locally-made varieties of unlabeled, unrecorded spirits are legal, inexpensive and readily available, especially in rural areas [17]. Medicinal spirits (distilled spirits compounded with plant and animal ingredients) are part of Chinese traditional medicinal. In addition, there is little understanding of how much wine is used in traditional Chinese cooking, and whether the cooking methods ensure that the alcohol is fully reduced before the food is eaten. To accommodate this wider range of beverage alcohols, some large surveys of Chinese alcohol use have included questions on as many as five beverage alcohol categories (but not medicinal spirits) [3, 18].

#### Quantity

Alcohol survey questions ask if a respondent has drunk alcohol at least once “including one sip,” some questions specify “at least one cup.” There is an absence of a widely accepted understanding of a “standard drink” both in terms of the drinking cup size and the beverage container size. Beer is sold in reasonably uniform-sized containers, ranging from 300 ml to 1 L, so survey data on beer consumption can probably provide useful information on quantity. Fruit wine (especially imported wine) is often in standard bottles and served in wine glasses; however, wine currently makes up less than 3% of alcohol consumed in China [2]. Spirits are sold in containers of varied sizes. Spirits are often served in cups, glasses and bowls of different sizes and shapes that are frequently topped up by others in the drinking group, making it difficult to estimate quantity consumed. Even a simple survey question defining alcohol as “more than one cup” can be interpreted many ways. In terms of trying to estimate alcohol consumption in terms of liters of pure alcohol, it should be noted that Chinese spirits come in a wide range of strengths.

#### Translating survey questions

Translating Western alcohol survey questions is challenging, and the accuracy of the translation directly affects the validity of the data collected. There are many Chinese words for different types of alcohol. In everyday conversation “wine” (*jiu*, 酒) is used as a generic term for all types of alcohol. Beer is sometimes not thought of, by survey respondents, as a form of alcohol. Because alcohol use, even by very young people, is an unremarkable event, especially at festivals, unless carefully specified in the questionnaire many drinking events may be overlooked by respondents when answering questions about alcohol use.

These wide-ranging concerns about questionnaire development may explain the differences in rates reported in studies that used investigator-developed questionnaires and studies that used YRBS-based questionnaires.

### Geographic Region

The moderator geographic region (east vs. central, east vs. west) was associated with the logit drinking rates for male university students. Surveys of students at central China universities reported higher drinking rates. Surveys of students at eastern China universities reported lower drinking rates, and surveys in western China reported drinking rates in between.

A meta-analysis of studies of high school student alcohol use also found higher drinking rates were reported from surveys of west China adolescents. That meta-analysis of high school surveys found surveys from central China reported lower drinking rates, and surveys from eastern China were in between [10]. Neither the meta-analysis of high school drinking studies [10] or this meta-analysis of university drinking studies showed any significant north–south differences.

Because high schools enroll local adolescents, we expected to find some geographic differences that reflected regional differences in alcohol use. Universities, on the other hand, especially the more prestigious universities, serve students from all parts of the country, so accounting for differences due to the geographic region of the survey is more difficult.

The very suggestion of drinking rates varying at universities in different regions of the country begs exploration, as does the effect of geographic region on male drinking but not on female drinking. Geographic differences in drinking rates in a country as large and diverse as China calls into question the usefulness of national drinking rate estimates in alcohol policy development.

### Limitations

Many technical issues, not assessed in this analysis, could have affected the results. Survey research is relatively new in China and surveys of populations like university students are often conducted by well-meaning investigators with little experience. We have no information on the conditions under which the data were gathered, how confidentiality was assured, how any assurance of anonymity was interpreted or the methods used for data collection. We have no information on how the samples were identified and how individuals who completed surveys were recruited. We have no information on the times of the year the surveys were conducted. Timing could have affected survey results if data were collected within 30 days of significant festivals, rites of passage and other significant events in which alcohol is a traditional part. The diversity of the students in the samples represented in these papers may not have been truly representative of the Chinese university student population. We know little about data management and analysis techniques used in the studies included in this analysis.

## Conclusions

This meta-analysis of 30 studies published in Chinese and English between 2006 and 2015 of self-reported alcohol use by Chinese undergraduate university students (approximate age 18–23 years old) provided an estimate alcohol use in the last 30 days of 66.8% for males and 31.7% for females.

## Abbreviations

ANOVA: Analysis of variance
CNKI: China National Knowledge Infrastructure (database)
PM: PubMed (database)
WF: Wanfang (database)
WHO: World Health Organization
WS: Web of Science (database)
YRBS: Youth Risk Behavior Surveillance Survey
